# Exploring the genetics of lithium response in bipolar disorders

**DOI:** 10.1186/s40345-024-00341-y

**Published:** 2024-06-12

**Authors:** Marisol Herrera-Rivero, Mazda Adli, Kazufumi Akiyama, Nirmala Akula, Azmeraw T. Amare, Raffaella Ardau, Bárbara Arias, Jean-Michel Aubry, Lena Backlund, Frank Bellivier, Antonio Benabarre, Susanne Bengesser, Abesh Kumar Bhattacharjee, Joanna M. Biernacka, Armin Birner, Micah Cearns, Pablo Cervantes, Hsi-Chung Chen, Caterina Chillotti, Sven Cichon, Scott R. Clark, Francesc Colom, Cristiana Cruceanu, Piotr M. Czerski, Nina Dalkner, Franziska Degenhardt, Maria Del Zompo, J. Raymond DePaulo, Bruno Etain, Peter Falkai, Ewa Ferensztajn-Rochowiak, Andreas J. Forstner, Josef Frank, Louise Frisén, Mark A. Frye, Janice M. Fullerton, Carla Gallo, Sébastien Gard, Julie S. Garnham, Fernando S. Goes, Maria Grigoroiu-Serbanescu, Paul Grof, Ryota Hashimoto, Roland Hasler, Joanna Hauser, Urs Heilbronner, Stefan Herms, Per Hoffmann, Liping Hou, Yi-Hsiang Hsu, Stephane Jamain, Esther Jiménez, Jean-Pierre Kahn, Layla Kassem, Tadafumi Kato, John Kelsoe, Sarah Kittel-Schneider, Po-Hsiu Kuo, Ichiro Kusumi, Barbara König, Gonzalo Laje, Mikael Landén, Catharina Lavebratt, Marion Leboyer, Susan G. Leckband, Mario Maj, Mirko Manchia, Cynthia Marie-Claire, Lina Martinsson, Michael J. McCarthy, Susan L. McElroy, Vincent Millischer, Marina Mitjans, Francis M. Mondimore, Palmiero Monteleone, Caroline M. Nievergelt, Tomas Novák, Markus M. Nöthen, Claire O’Donovan, Norio Ozaki, Sergi Papiol, Andrea Pfennig, Claudia Pisanu, James B. Potash, Andreas Reif, Eva Reininghaus, Hélène Richard-Lepouriel, Gloria Roberts, Guy A. Rouleau, Janusz K. Rybakowski, Martin Schalling, Peter R. Schofield, Klaus Oliver Schubert, Eva C. Schulte, Barbara W. Schweizer, Giovanni Severino, Tatyana Shekhtman, Paul D. Shilling, Katzutaka Shimoda, Christian Simhandl, Claire M. Slaney, Alessio Squassina, Thomas Stamm, Pavla Stopkova, Fabian Streit, Fasil Tekola-Ayele, Anbupalam Thalamuthu, Alfonso Tortorella, Gustavo Turecki, Julia Veeh, Eduard Vieta, Biju Viswanath, Stephanie H. Witt, Peter P. Zandi, Martin Alda, Michael Bauer, Francis J. McMahon, Philip B. Mitchell, Marcella Rietschel, Thomas G. Schulze, Bernhard T. Baune

**Affiliations:** 1 Department of Psychiatry, University of Münster and Joint Institute for Individualisation in a Changing Environment (JICE), University of Münster and Bielefeld University, Albert-Schweitzer-Campus 1, Building A9, 48149 Münster, Germany; 2 Department of Psychiatry and Psychotherapy, Charité, Universitätsmedizin Berlin, Campus Charité Mitte, Berlin, Germany; 3 Fliedner Klinik Berlin, Berlin, Germany; 4 Department of Biological Psychiatry and Neuroscience, Dokkyo Medical University School of Medicine, Mibu, Japan; 5 Intramural Research Program, National Institute of Mental Health, National Institutes of Health, US Department of Health & Human Services, Baltimore, USA; 6 Discipline of Psychiatry, School of Medicine, University of Adelaide, Adelaide, SA Australia; 7 Unit of Clinical Pharmacology, Hospital University Agency of Cagliari, Cagliari, Italy; 8 Unitat de Zoologia i Antropologia Biològica (Dpt. Biologia Evolutiva, Ecologia i Ciències Ambientals), Facultat de Biologia and Institut de Biomedicina (IBUB), University of Barcelona, CIBERSAM, Barcelona, Spain; 9 Department of Psychiatry, Division of Psychiatric Specialities, Geneva University Hospitals, Geneva, Switzerland; 10 Faculty of Medicine, University of Geneva, Geneva, Switzerland; 11 Department of Molecular Medicine and Surgery and Center for Molecular Medicine at Karolinska University Hospital, Karolinska Institute, Stockholm, Sweden; 12 Département de Psychiatrie et de Médecine Addictologique, INSERM UMR-S 1144, Université Paris Cité, AP-HP, Groupe Hospitalier Saint-Louis-Lariboisière, F. Widal, Paris, France; 13 Bipolar Disorder Program, Institute of Neuroscience, Hospital Clinic, University of Barcelona, IDIBAPS, CIBERSAM, Barcelona, Spain; 14 Department of Psychiatry and Psychotherapeutic Medicine, Research Unit for Bipolar Affective Disorder, Medical University of Graz, Graz, Austria; 15 Department of Psychiatry, University of California San Diego, San Diego, USA; 16 Department of Health Sciences Research, Mayo Clinic, Rochester, USA; 17 Department of Psychiatry and Psychology, Mayo Clinic, Rochester, USA; 18 The Neuromodulation Unit, McGill University Health Centre, Montreal, Canada; 19 Department of Psychiatry & Center of Sleep Disorders, National Taiwan University Hospital, Taipei, Taiwan; 20 Human Genomics Research Group, Department of Biomedicine, University Hospital Basel, Basel, Switzerland; 21 Institute of Medical Genetics and Pathology, University Hospital Basel, Basel, Switzerland; 22 Institute of Neuroscience and Medicine (INM-1), Research Center Jülich, Jülich, Germany; 23 Mental Health Research Group, IMIM-Hospital del Mar, Barcelona, Spain; 24 Centro de Investigación Biomédica en Red de Salud Mental (CIBERSAM), Instituto de Salud Carlos III, Madrid, Spain; 25 Douglas Mental Health University Institute, McGill University, Montreal, Canada; 26 Psychiatric Genetic Unit, Poznan University of Medical Sciences, Poznań, Poland; 27 Institute of Human Genetics, University of Bonn, School of Medicine & University Hospital Bonn, Bonn, Germany; 28 Department of Biomedical Sciences, University of Cagliari, Cagliari, Italy; 29 Department of Psychiatry and Behavioral Sciences, Johns Hopkins University, Baltimore, USA; 30 Department of Psychiatry and Psychotherapy, Ludwig-Maximilian-University Munich, Munich, Germany; 31 Department of Adult Psychiatry, Poznan University of Medical Sciences, Poznań, Poland; 32 Department of Genetic Epidemiology in Psychiatry, Central Institute of Mental Health, Medical Faculty Mannheim, University of Heidelberg, Heidelberg, Germany; 33 Centre for Psychiatry Research, Department of Clinical Neuroscience, Karolinska Institutet, Stockholm, Sweden; 34 Neuroscience Research, Australia and School of Biomedical Sciences, University of New South Wales, Sydney, Australia; 35 Laboratorios de Investigación y Desarrollo, Facultad de Ciencias y Filosofía, Universidad Peruana Cayetano Heredia, San Martín de Porres, Peru; 36 Service de Psychiatrie, Hôpital Charles Perrens, Bordeaux, France; 37 Department of Psychiatry, Dalhousie University, Halifax, Canada; 38 Biometric Psychiatric Genetics Research Unit, Alexandru Obregia Clinical Psychiatric Hospital, Bucharest, Romania; 39 Mood Disorders Center of Ottawa, Ottawa, Canada; 40 Department of Pathology of Mental Diseases, National Institute of Mental Health, National Center of Neurology and Psychiatry, Tokyo, Japan; 41 Institute of Psychiatric Phenomics and Genomics (IPPG), University Hospital, LMU Munich, Munich, Germany; 42 Program for Quantitative Genomics, Harvard School of Public Health and HSL Institute for Aging Research, Harvard Medical School, Boston, USA; 43 Univ. Paris Est Créteil, INSERM, IMRB, Translational Neuropsychiatry, Fondation FondaMental, Créteil, France; 44 Bipolar and Depressive Disorders Unit, Institute of Neuroscience, Hospital Clinic, University of Barcelona, IDIBAPS, CIBERSAM, ISCIII, Barcelona, Spain; 45 Service de Psychiatrie et Psychologie Clinique, Centre Psychothérapique de Nancy - Université, Nancy, France; 46 Department of Psychiatry & Behavioral Science, Graduate School of Medicine, Juntendo University, Tokyo, Japan; 47 Department of Psychiatry, Psychosomatic Medicine and Psychotherapy, University Hospital Würzburg, Würzburg, Germany; 48 Department of Public Health & Institute of Epidemiology and Preventive Medicine, College of Public Health, National Taiwan University, Taipei, Taiwan; 49 Department of Psychiatry, Hokkaido University Graduate School of Medicine, Sapporo, Japan; 50 Department of Psychiatry and Psychotherapeutic Medicine, Landesklinikum Neunkirchen, Neunkirchen, Austria; 51 Institute of Neuroscience and Physiology, The Sahlgrenska Academy at the Gothenburg University, Gothenburg, Sweden; 52 Department of Medical Epidemiology and Biostatistics, Karolinska Institutet, Stockholm, Sweden; 53 Univ. Paris Est Créteil, INSERM, IMRB, Translational Neuropsychiatry, AP-HP, Mondor University Hospital, DMU Impact, Fondation FondaMental, Créteil, France; 54 Office of Mental Health, VA San Diego Healthcare System, California, USA; 55 Department of Psychiatry, University of Campania ‘Luigi Vanvitelli’, Caserta, Italy; 56 Section of Psychiatry, Department of Medical Sciences and Public Health, University of Cagliari, Cagliari, Italy; 57 Department of Pharmacology, Dalhousie University, Halifax, Canada; 58 Université Paris Cité, Inserm UMR-S 1144, Optimisation Thérapeutique en Neuropsychopharmacologie, 75006 Paris, France; 59 Department of Clinical Neurosciences, Karolinska Institutet, Stockholm, Sweden; 60 Department of Psychiatry, VA San Diego Healthcare System, San Diego, CA USA; 61 Department of Psychiatry, Lindner Center of Hope/University of Cincinnati, Cincinnati, USA; 62 Department of Psychiatry and Psychotherapy, Comprehensive Center for Clinical Neurosciences and Mental Health, Medical University of Vienna, Vienna, Austria; 63 Department of Genetics, Microbiology and Statistics, Faculty of Biology, Institut de Biomedicina de La Universitat de Barcelona (IBUB), University of Barcelona, Barcelona, Spain; 64 Department of Medicine, Surgery and Dentistry ‘Scuola Medica Salernitana’, University of Salerno, Baronissi, Italy; 65 National Institute of Mental Health, Klecany, Czech Republic; 66 Department of Psychiatry & Department of Child and Adolescent Psychiatry, Nagoya University Graduate School of Medicine, Nagoya, Japan; 67 Department of Psychiatry and Psychotherapy, University Hospital Carl Gustav Carus, Medical Faculty, Technische Universität Dresden, Dresden, Germany; 68 Department of Psychiatry, Psychosomatic Medicine and Psychotherapy, University Hospital Frankfurt, Frankfurt, Germany; 69 School of Psychiatry, University of New South Wales, Sydney, Australia; 70 Montreal Neurological Institute and Hospital, McGill University, Montreal, Canada; 71 Northern Adelaide Local Health Network, Mental Health Services, Adelaide, Australia; 72 Department of Psychiatry and Psychotherapy, University Hospital Bonn, Medical Faculty University of Bonn, Bonn, Germany; 73 Department of Psychiatry, Dokkyo Medical University School of Medicine, Mibu, Japan; 74 Medical Faculty, Bipolar Center Wiener Neustadt, Sigmund Freud University, Vienna, Austria; 75 Epidemiology Branch, Division of Intramural Population Health Research, Eunice Kennedy Shriver National Institute of Child Health and Human Development, National Institutes of Health, Bethesda, USA; 76 Centre for Healthy Brain Ageing (CHeBA), School of Psychiatry, University of New South Wales, Sydney, Australia; 77 Department of Psychiatry, University of Perugia, Perugia, Italy; 78 Department of Psychiatry, National Institute of Mental Health and Neurosciences, Bangalore, 560029 India; 79 Department of Mental Health, Johns Hopkins Bloomberg School of Public Health, Baltimore, USA; 80 Department of Psychiatry and Behavioral Sciences, Norton College of Medicine, SUNY Upstate Medical University, Syracuse, NY USA; 81 Department of Psychiatry, Melbourne Medical School, University of Melbourne and The Florey Institute of Neuroscience and Mental Health, The University of Melbourne, Melbourne, Australia

**Keywords:** Bipolar disorder, Lithium treatment, Psychiatric symptoms, Comorbidity, Genetics

## Abstract

**Background:**

Lithium (Li) remains the treatment of choice for bipolar disorders (BP). Its mood-stabilizing effects help reduce the long-term burden of mania, depression and suicide risk in patients with BP. It also has been shown to have beneficial effects on disease-associated conditions, including sleep and cardiovascular disorders. However, the individual responses to Li treatment vary within and between diagnostic subtypes of BP (e.g. BP-I and BP-II) according to the clinical presentation. Moreover, long-term Li treatment has been linked to adverse side-effects that are a cause of concern and non-adherence, including the risk of developing chronic medical conditions such as thyroid and renal disease. In recent years, studies by the Consortium on Lithium Genetics (ConLiGen) have uncovered a number of genetic factors that contribute to the variability in Li treatment response in patients with BP. Here, we leveraged the ConLiGen cohort (N = 2064) to investigate the genetic basis of Li effects in BP. For this, we studied how Li response and linked genes associate with the psychiatric symptoms and polygenic load for medical comorbidities, placing particular emphasis on identifying differences between BP-I and BP-II.

**Results:**

We found that clinical response to Li treatment, measured with the Alda scale, was associated with a diminished burden of mania, depression, substance and alcohol abuse, psychosis and suicidal ideation in patients with BP-I and, in patients with BP-II, of depression only. Our genetic analyses showed that a stronger clinical response to Li was modestly related to lower polygenic load for diabetes and hypertension in BP-I but not BP-II. Moreover, our results suggested that a number of genes that have been previously linked to Li response variability in BP differentially relate to the psychiatric symptomatology, particularly to the numbers of manic and depressive episodes, and to the polygenic load for comorbid conditions, including diabetes, hypertension and hypothyroidism.

**Conclusions:**

Taken together, our findings suggest that the effects of Li on symptomatology and comorbidity in BP are partially modulated by common genetic factors, with differential effects between BP-I and BP-II.

**Supplementary Information:**

The online version contains supplementary material available at 10.1186/s40345-024-00341-y.

## Background

Lithium (Li) is the first-line maintenance treatment for bipolar disorders (BP). Multiple beneficial properties have been attributed to Li, including mood stabilization, cardio- and neuroprotection, circadian regulation, immunomodulation, and suicide prevention in patients with BP (Geoffroy et al. 2016; Volkmann et al. 2020; Xu et al. 2021; Queissner et al. 2021; Miller & McCall 2022; Rybakowski 2022; Chen et al. 2023; Szałach et al. 2023). Li is not exempt from acute side-effects, the most frequent being gastrointestinal complaints, that may cause non-adherence. However, it is the long-term adverse effects, including thyroid and kidney problems (Volkmann et al. 2020; Ferensztajn-Rochowiak et al. 2021), that cause most concern.

Individual responses to Li vary according to the clinical presentation of the disease. Reportedly, only about 30% of patients with BP have a full response to Li treatment. Various clinical, psychosocial and demographic factors that affect Li response have been described (Nunes et al. 2020; Ferensztajn-Rochowiak et al. 2021). Moreover, genetic studies have established Li response as a polygenic trait (Papiol et al. 2022). Previous work performed by the Consortium on Lithium Genetics (ConLiGen) has offered significant insights into the molecular mechanisms contributing to Li response (Amare et al. 2023), as well as the links with the polygenic scores of other psychiatric disorders (Amare et al. 2018; Schubert et al. 2021; Coombes et al. 2021) and with suicidal behavior (Yoshida et al. 2019) in BP. However, the relationships between Li response and disease features, particularly comorbidity, remain largely unexplored. Moreover, most studies have made no distinction between different diagnostic groups. Here, we used data from ConLiGen participants (N = 2064) to explore how the genetic factors that contribute to Li response variability in patients with BP are associated with specific psychiatric symptoms and the polygenic load (i.e. genetic risk) for medical comorbid conditions, and whether these relationships differ between BP types I and II.

## Methods

### Study population

The ConLiGen cohort has been described elsewhere (Hou et al. 2016). Briefly, between 2003 and 2013, ConLiGen recruited over 2500 Li-treated individuals with bipolar spectrum disorders at various sites in Europe, the United States, Australia and East-Asia. The inclusion criteria consisted of a diagnosis of bipolar disorder type I (BP-I) or type II (BP-II), schizoaffective bipolar disorder or bipolar disorder not otherwise specified in accordance with the criteria established in the Diagnostic and Statistical Manual of Mental Disorders (DSM) versions III or IV, as well as Li treatment that lasted a minimum of six months with no additional mood stabilizers. Long-term responses to Li treatment were assessed using the Alda scale, where an A subscale rates the degree of response in the range 0–10 and a B subscale reflects the relationship between improvement and treatment. A total score, ranging from 0–10, is obtained by subtracting the B score from the A score (Manchia et al. 2013). Negative scores are set to 0. Here, we used a sample of 2064 ConLiGen participants with complete covariate phenotypes: sex, age-at-onset (AAO), age at recruitment (i.e. sample collection), diagnosis and recruitment site (used to establish population).

The Ethics Committee at the University of Heidelberg provided central approval for ConLiGen. Written informed consent from all participants was obtained according to the study protocols of each of the participating sites and their institutions. All procedures were performed in accordance with the guidelines of the Declaration of Helsinki.

### Genotype data

Genotyping, quality control (QC) and imputation of the ConLiGen cohort has been described elsewhere (Hou et al. 2016). Briefly, DNA genotyping by array was performed from peripheral blood samples in two batches of similar composition, originally referred to as “GWAS1” (N = 1162) and “GWAS2” (N = 1401). Standard procedures for QC and imputation using the 1000 Genomes Project reference panel were employed. Here, we used an updated ConLiGen dataset we previously described in detail (Herrera-Rivero et al. 2024), in which we re-imputed the combined ConLiGen batches using the Haplotype Reference Consortium (HRC) panel. This procedure increased the number of markers and the quality of the dataset, increasing its suitability for polygenic score (PGS) analyses. Single nucleotide polymorphisms (SNPs) in 37 genes that were previously reported to contribute to Li response in ConLiGen following a gene-level genome-wide analysis (Amare et al. 2023) were extracted from the dataset using a window of ± 1 kb from the start and end positions of the gene (according to the Ensembl hg19 genome build). Our final dataset contained 9374 SNPs corresponding to 34 Li response-linked genes and 2064 individuals with BP, from which 1669 had a diagnosis of BP-I and 370 of BP-II.

### Phenotypes

#### Li response

We used the total Alda score as a measure of Li response. This was available for all 2064 individuals included in our study.

#### Psychiatric symptoms

Here, the psychiatric symptoms corresponded to the numbers of episodes of depression and mania, the presence of psychosis, alcohol and substance abuse, and of suicidal ideation. These variables were available for a maximum of 853 individuals from the GWAS1 batch.

#### Genetic risk for medical comorbidities

Based on the literature, we identified various conditions that are comorbid in BP and searched the PGS Catalog (Lambert et al. 2021) for publicly available PGSs for these. Weight files for the calculation of PGSs for various traits, such as disorders of sleep and metabolism, were downloaded from the PGS Catalog and used for allelic scoring in the total ConLiGen sample with plink 1.9 (Chang et al. 2015). Standardized sum scores were used for analysis. Because of incomplete compatibility between PGS SNPs and variants in the ConLiGen dataset, only PGSs with compatibility > 78% were used. These corresponded to the following traits: chronotype (PGS ID: PGS002209), sleep duration (PGS ID: PGS002196), insomnia (PGS ID: PGS002149), hypertension (PGS ID: PGS002047), hypothyroidism (PGS ID: PGS001816) and type 2 diabetes (PGS ID: PGS003118) (Privé et al. 2022; Ma et al. 2022) (Suppl.Table 1). Traits excluded due to lower compatibility included cardiovascular disorders, obesity, migraine and asthma.

### Statistical analyses

Associations between total Alda scores and psychiatric symptoms were tested using robust linear/logistic regression models with the “robustbase” R package (n_max_ = 853). Models were adjusted for sex, AAO and age. Associations between total Alda scores and PGSs for comorbid conditions were tested using partial Spearman correlation with the “ppcor” R package (n_max_ = 2064). Models were adjusted for sex, AAO, age and population. SNP-phenotype associations were tested using linear/logistic regression models with plink 1.9. Models were adjusted for sex, AAO, age, population, total Alda score and the first eight dimensions coming from a principal components analysis of the genotypes. When testing associations using all individuals, all models were also adjusted for the differential BP diagnosis. All associations were also tested separately for BP-I and BP-II. For exploratory purposes, significance was set to nominal (i.e. unadjusted) p < 0.05 and p < 0.01 for total Alda score and SNP-phenotype associations, respectively.

## Results

To explore how Li response genes are associated with specific psychiatric symptoms and the poygenic load for medical comorbid conditions, and whether these relationships differ between BP types I and II, we used a sample of 2064 individuals with BP from the ConLiGen cohort. From these, 1197 (58%) were females, 1669 (80.1%) had a diagnosis of BP-I and 370 (17.9%) were diagnosed with BP-II. The mean AAO in the sample was 25 ± 11 years, while the mean age at recruitment was 47 ± 14 years. The mean total Alda score was 4.22 ± 3.16 points, with 29.8% of the patients being categorized as good responders (total Alda score ≥ 7). Compared to BP-I, BP-II patients were slightly older at disease onset (28 ± 12 vs 24 ± 10 years) and recruitment (50 ± 14 vs 47 ± 14 years), and had higher rates of females (61.9% vs 57.2%) and good Li responders (34.1% vs 28.2%). However, the mean total Alda scores were very similar (4.6 ± 3.2 vs 4.2 ± 3.1 points).

**Fig. 1:**
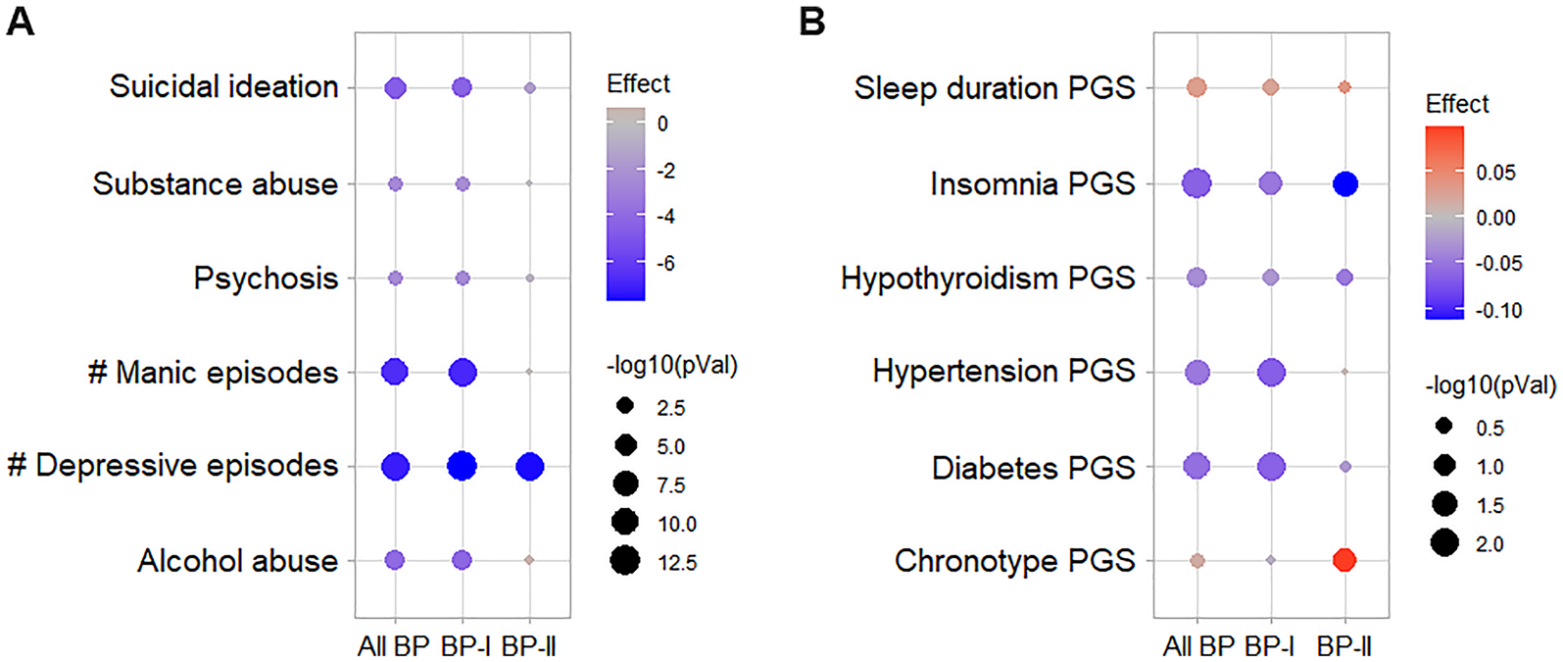
Links between phenotypes and Li responses in ConLiGen. **A** Association test results between total Alda scores and psychiatric symptoms. Shown are the nominal p-values (−log10) and z-values (effect) obtained from robust linear/logistic regression models. **B** Correlation test results between total Alda scores and PGSs for comorbid conditions. Shown are the nominal p-values (−log10) and correlation coefficients (effect) obtained from partial correlation models using the Spearman method

First, we explored the association between Li response and psychiatric symptoms/PGSs for comorbid conditions. Using a nominal significance threshold (p < 0.05), we found that the total Alda scores showed a negative relationship with all psychiatric symptom variables in all BP (n_max_ = 835) and BP-I (n_max_ = 665) individuals. However, in BP-II individuals (n_max_ = 153), the total Alda scores showed a negative relationship only with the number of depressive episodes (Fig. 1A). Noticeably, these results survived false discovery rate correction (FDR < 0.05). Furthermore, the total Alda scores also correlated negatively with the PGSs for diabetes and hypertension in all BP (N = 2064) and BP-I (N = 1669) individuals, and with the PGS for insomnia in all BP, BP-I and BP-II (N = 370) individuals (Fig. 1B). However, none of the nominal associations with PGSs survived FDR correction in our sample.

**Fig. 2:**
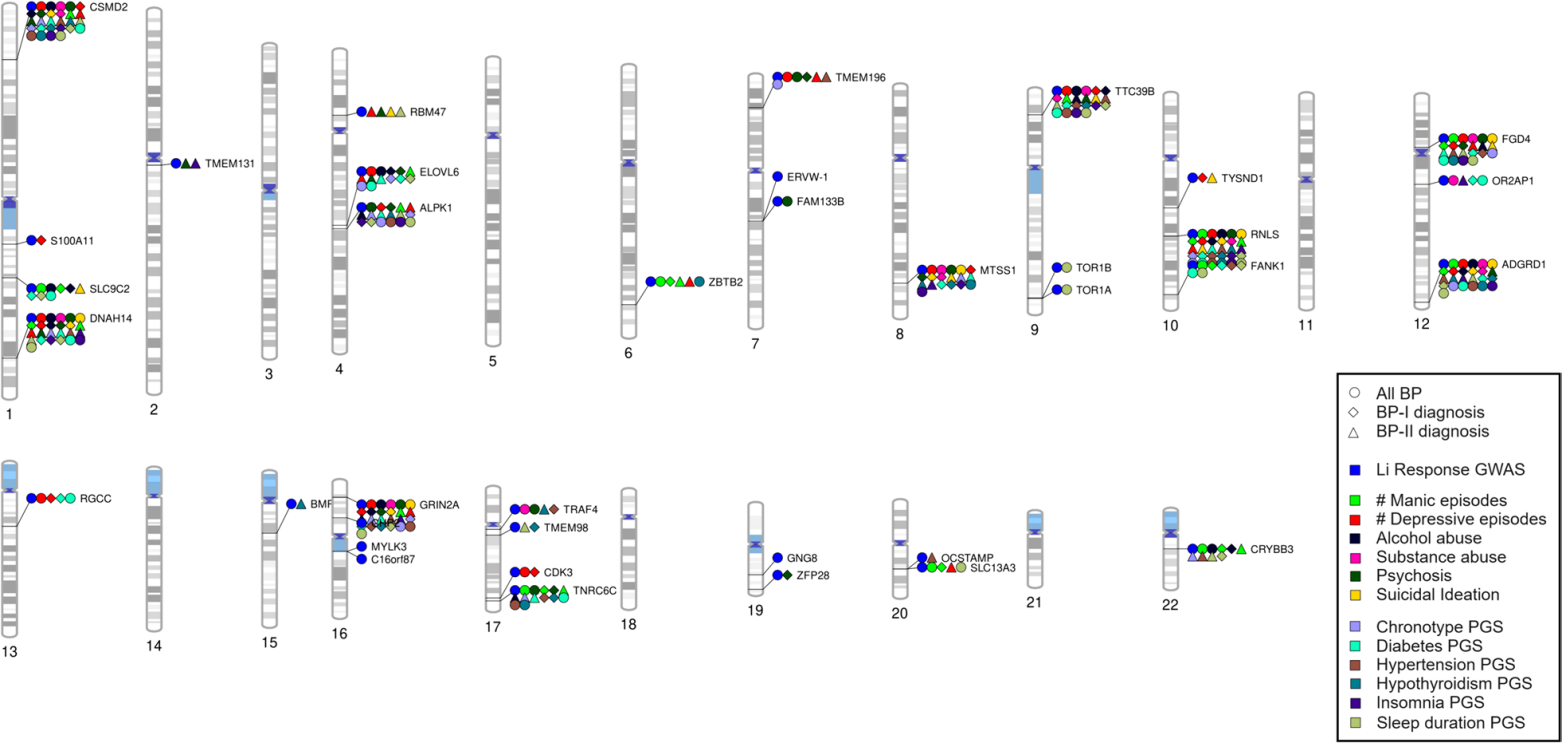
Visual integration of nominal findings for Li response genes. Shapes depict the diagnostic group analyzed while colors refer to the phenotypes nominally associated with the gene in our analyses, except for the blue color, which localized even the genes not analyzed in this study that were reported by Amare et al. 2023 as contributors to Li response in ConLiGen

Second, we explored the association between genes previously linked to Li response and psychiatric symptoms/PGSs for comorbid conditions. Using a nominal significance threshold (p < 0.01) as indicative of suggestive association, we found that 32 of the 34 genes tested were suggested to associate with specific psychiatric symptoms and/or PGSs for comorbid conditions (Fig. 2, Suppl.Tables.2–7). The most significant hits were for the number of manic episodes, with *SLC13A3* as top gene in BP-I and *TNRC6C* in BP-II, followed by the number of depressive episodes, with *MTSS1* as top gene in BP-I and *DNAH14* in BP-II (Table 1).

**Table 1:** Phenotype-based summary of findings for the association analyses between Li response genes and psychiatric symptoms/PGSs for comorbid conditions in ConLiGen

Phenotype	N	# Cases	# Controls	# SNPs p < 0.01	# Genes	Top gene	Top # SNPs p < 0.01	Lowest p
*All BP*								
# Manic episodes	724	–	–	38	9	*SLC13A3*	11	2.48E−08
# Depressive episodes	789	–	–	225	12	*FGD4*	75	5.15E−06
Alcohol abuse	835	140	695	114	9	*ELOVL6*	5	1.11E−04
Substance abuse	832	135	697	143	9	*ADGRD1*	45	4.17E−04
Psychosis	692	342	350	55	11	*GRIN2A*	12	7.83E−04
Suicidal ideation	660	321	339	10	6	*DNAH14*	1	2.31E−03
Insomnia PGS	2064	–	–	57	8	*CSMD2*	6	1.73E−04
Sleep duration PGS	2064	–	–	211	12	*DNAH14*	133	1.12E−04
Chronotype PGS	2064	–	–	81	7	*GRIN2A*	47	4.06E−04
Diabetes PGS	2064	–	–	111	12	*CSMD2*	33	6.28E−04
Hypertension PGS	2064	–	–	34	7	*TTC39B*	5	9.57E−05
Hypothyroidism PGS	2064	–	–	82	7	*MTSS1*	42	4.73E−04
*BP-I diagnosis*								
# Manic episodes	641	–	–	48	10	*SLC13A3*	11	2.15E−08
# Depressive episodes	632	–	–	193	13	*MTSS1*	12	1.52E−06
Alcohol abuse	665	129	536	131	9	*CSMD2*	52	1.34E−04
Substance abuse	662	121	541	121	5	*ADGRD1*	52	4.13E−04
Psychosis	564	318	246	87	10	*CSMD2*	21	7.17E−04
Suicidal ideation	530	264	266	41	6	*MTSS1*	1	2.15E−04
Insomnia PGS	1669	–	–	48	6	*ALPK1*	4	3.92E−04
Sleep duration PGS	1669	–	–	174	11	*RNLS*	3	4.37E−05
Chronotype PGS	1669	–	–	35	5	*RNLS*	2	1.76E−04
Diabetes PGS	1669	–	–	74	13	*TTC39B*	1	6.78E−04
Hypertension PGS	1669	–	–	29	7	*TTC39B*	1	6.81E−04
Hypothyroidism PGS	1669	–	–	38	8	*CSMD2*	4	6.95E−04
*BP-II diagnosis*								
# Manic episodes	68	–	–	113	10	*TNRC6C*	3	3.76E−79
# Depressive episodes	141	–	–	128	11	*DNAH14*	6	3.12E−08
Alcohol abuse	153	7	146	7	5	*TNRC6C*	2	1.80E−03
Substance abuse	153	8	145	0	0	–	–	–
Psychosis	115	12	103	353	7	*TMEM131*	46	1.08E−03
Suicidal ideation	118	48	70	79	7	*TTC39B*	24	2.49E−03
Insomnia PGS	370	–	–	209	7	*GRIN2A*	38	2.65E−04
Sleep duration PGS	370	–	–	64	9	*DNAH14*	16	2.95E−04
Chronotype PGS	370	–	–	32	7	*GRIN2A*	19	1.81E−03
Diabetes PGS	370	–	–	97	9	*MTSS1*	6	2.01E−04
Hypertension PGS	370	–	–	130	10	*TMEM196*	27	3.21E−04
Hypothyroidism PGS	370	–	–	70	7	*BMF*	12	1.92E−04

Taken together, 22 of the 34 genes tested were nominally associated with at least one psychiatric symptom and one PGS in at least one of the tests performed (i.e. all BP, BP-I and BP-II). Noticeably, some of the Li response genes were suggested to associate with all the phenotypes that we studied in at least one of the tests. We also observed that genes with the most overlaps, including *RNLS*, *GRIN2A*, *CSMD2*, *DNAH14* and *TTC39B* (Table 2), represented the most significant hits obtained in BP-I or BP-II for various PGSs for comorbid conditions (Table 1).

**Table 2:** Gene-based summary of findings for the association analyses between Li response genes and psychiatric symptoms/PGSs for comorbid conditions in ConLiGen

Gene	Chr	Gene start (− 1 kb)	Gene end (+ 1 kb)	# tested SNPs	Psychiatric phenotype count			PGS phenotype count			Max. # phenotypes
					All	BP-I	BP-II	All	BP-I	BP-II	
*CSMD2*	1	33,978,609	34,632,443	1064	4	5	3	5	5	5	12
*S100A11*	1	152,003,982	152,021,383	14	0	1	0	0	0	0	1
*SLC9C2*	1	173,468,603	173,573,233	179	2	2	1	1	2	0	5
*DNAH14*	1	225,082,964	225,587,996	1417	5	5	3	3	3	5	11
*TMEM131*	2	98,371,799	98,613,388	358	0	0	1	0	0	1	2
*RBM47*	4	40,424,272	40,633,892	164	0	0	3	0	0	1	4
*ELOVL6*	4	110,966,002	111,121,355	261	2	2	3	2	3	1	7
*ALPK1*	4	113,205,665	113,364,776	301	1	2	3	4	3	4	10
*ZBTB2*	6	151,684,252	151,713,683	43	1	1	2	1	0	0	3
*TMEM196*	7	19,757,933	19,814,221	108	2	1	1	1	0	1	4
*ERVW-1*	7	92,096,694	92,108,300	19	0	0	0	0	0	0	0
*FAM133B*	7	92,189,107	92,220,708	50	1	0	0	0	0	0	1
*MTSS1*	8	125,562,031	125,741,730	499	4	4	1	2	3	4	8
*TTC39B*	9	15,162,620	15,308,358	408	3	3	4	4	5	2	11
*TOR1B*	9	132,564,432	132,574,560	20	0	0	0	1	0	0	1
*TOR1A*	9	132,574,223	132,587,413	32	0	0	0	1	0	0	1
*TYSND1*	10	71,896,737	71,907,432	40	0	1	1	0	0	0	2
*RNLS*	10	90,032,621	90,345,287	628	5	5	3	6	6	4	12
*FANK1*	10	127,584,108	127,699,161	250	1	1	0	2	3	0	4
*FGD4*	12	32,551,463	32,799,984	882	5	3	3	5	2	3	12
*OR2AP1*	12	55,967,199	55,970,128	7	1	0	0	1	1	1	3
*ADGRD1*	12	131,437,452	131,627,014	603	5	5	1	6	3	4	12
*RGCC*	13	42,030,695	42,046,018	35	1	1	0	1	1	0	2
*BMF*	15	40,379,091	40,402,093	16	0	0	0	0	0	1	1
*GRIN2A*	16	9,851,376	10,277,611	1624	5	4	3	3	5	4	12
*CHP2*	16	23,764,948	23,771,272	10	0	0	0	0	0	0	0
*MYLK3*	16	46,739,891	46,825,319	0	0	0	0	0	0	0	0
*C16orf87*	16	46,829,519	46,866,323	0	0	0	0	0	0	0	0
*TRAF4*	17	27,070,002	27,078,974	8	2	0	0	0	1	1	4
*TMEM98*	17	31,253,928	31,273,124	33	0	0	0	0	1	1	2
*CDK3*	17	73,995,987	74,003,080	4	1	1	0	0	0	0	1
*TNRC6C*	17	75,999,249	76,105,916	153	2	2	2	3	2	2	7
*GNG8*	19	47,136,333	47,138,942	0	0	0	0	0	0	0	0
*ZFP28*	19	57,049,317	57,069,169	46	0	1	0	0	0	0	1
*OCSTAMP*	20	45,168,585	45,180,213	10	0	0	0	0	0	1	1
*SLC13A3*	20	45,185,463	45,305,714	58	1	1	1	1	0	0	3
*CRYBB3*	22	25,594,817	25,604,330	31	2	2	1	0	1	3	5

Finally, we looked into the overlapping and non-overlapping genes between the BP-I and BP-II analyses (Table 3). Here, we observed that, for example, *GRIN2A* was suggested to relate to the number of depressive episodes, the presence of alcohol abuse, and the polygenic contribution to chronotype, diabetes and hypertension in both major types of BP. However, it was suggested to be linked to the presence of psychosis and suicidal ideation, and the polygenic contribution to sleep duration and hypothyroidism in BP-I only, while relating to the number of manic episodes and the genetic load for insomnia only in BP-II.

**Table 3:** Li response genes nominally associated with psychiatric symptoms/PGSs for comorbid conditions in ConLiGen. Shown are the overlapping and non-overlapping genes between BP-I and BP-II diagnostic groups

Phenotype	BP-I only	BP-II only	Overlap
# Manic episodes	*ADGRD1, FANK1, FGD4, SLC13A3, SLC9C2*	*ALPK1, CSMD2, ELOVL6, GRIN2A, TTC39B*	*CRYBB3, DNAH14, RNLS, TNRC6C, ZBTB2*
# Depressive episodes	*ADGRD1, CDK3, MTSS1, RGCC, S100A11, TTC39B, TYSND1*	*ELOVL6, RBM47, SLC13A3, TMEM196, ZBTB2*	*ALPK1, CSMD2, DNAH14, FGD4, GRIN2A, RNLS*
Alcohol abuse	*ADGRD1, CRYBB3, CSMD2, DNAH14, ELOVL6, RNLS, SLC9C2*	*ALPK1, FGD4, TNRC6C*	*GRIN2A, TTC39B*
Substance abuse	*ADGRD1, CSMD2, MTSS1, RNLS, TTC39B*	*–*	*–*
Psychosis	*ALPK1, FGD4, GRIN2A, MTSS1, TMEM196, TNRC6C, ZFP28*	*ADGRD1, RBM47, TMEM131, TTC39B*	*CSMD2, DNAH14, ELOVL6*
Suicidal ideation	*ADGRD1, CSMD2, DNAH14, GRIN2A, MTSS1*	*FGD4, MTSS1, RBM47, SLC9C2, TTC39B, TYSND1*	*RNLS*
Insomnia PGS	*ALPK1, CSMD2, TTC39B*	*ADGRD1, GRIN2A, OR2AP1, TMEM131*	*DNAH14, MTSS1, RNLS*
Sleep duration PGS	*ELOVL6, FANK1, GRIN2A, RNLS, SLC9C2*	*FGD4, RBM47, TMEM98*	*ADGRD1, ALPK1, CRYBB3, CSMD2, DNAH14, TTC39B*
Chronotype PGS	*ELOVL6, RNLS*	*CRYBB3, DNAH14, MTSS1, TNRC6C*	*ALPK1, CSMD2, GRIN2A*
Diabetes PGS	*ADGRD1, FANK1, OR2AP1, SLC9C2, TTC39B*	*ALPK1, TNRC6C*	*CSMD2, DNAH14, ELOVL6, FGD4, GRIN2A, MTSS1, RNLS*
Hypertension PGS	*FANK1, TNRC6C, TRAF4*	*ADGRD1, CRYBB3, CSMD2, DNAH14, OCSTAMP, TMEM196*	*FGD4, GRIN2A, RNLS, TTC39B*
Hypothyroidism PGS	*GRIN2A, TMEM98, TNRC6C, TTC39B*	*ALPK1, BMF, TRAF4*	*ADGRD1, CSMD2, MTSS1, RNLS*

## Discussion

We showed that positive responses to Li treatment in patients with BP are generally more beneficial to those patients diagnosed with BP-I than to those with a BP-II diagnosis, and that genes linked to Li response also contribute to the clinical presentation of the disorder in terms of psychiatric symptomatology and, potentially, the risk of medical comorbid conditions. This may partly explain why Li responses usually vary according to clinical features, and why clinical and psychosocial factors can only partially predict Li responses (Tondo et al. 2001; Ferensztajn-Rochowiak et al. 2021).

Often, the efficacy of Li treatment in BP is assessed without making distinction between BP types and/or is focused on manic-depressive episodes, with disregard of other disease-associated afflictions. However, some studies have shown that Li impacts differently the frequency and duration of mood episodes in BP-I and BP-II (Tondo et al. 2001), which might relate to stronger effects on acute manic than depressive episodes (Fountoulakis et al. 2022). Moreover, it is plausible that the beneficial effects of Li treatment on psychiatric symptomatology are related to its effects on other health issues associated with BP, such as improving inflammation and sleep (Geoffroy et al. 2016; Szałach et al. 2023). The results of our study are in agreement. When we explored the association between Li response and psychiatric symptoms/PGSs for comorbid conditions, our observations suggested that better responses to Li treatment diminish the burden of most psychiatric symptoms in patients with BP-I, but only that of depression in patients with BP-II, and that better Li response differentially correlates with lower genetic burden predisposing to comorbid conditions, such as insomnia, diabetes and hypertension. In addition, when we explored the association between genes previously linked to Li response and psychiatric symptoms/PGSs for comorbid conditions, we found that Li response genes were more strongly associated with manic than depressive episodes in both BP-I and BP-II, and that Li response genes were modestly but differentially associated with other features relevant to the clinical presentation, including, for example, suicidal ideation, psychosis and polygenic load for insomnia and hypothyroidism, in both BP-I and BP-II. Noticeably, the fact that the results of our genetic analyses did not exactly match those obtained for the total Alda score, where the positive effects of Li showed a clear bias towards BP-I, also suggest important gene-environment interactions.

Despite the exploratory character of our genetic study, we believe that it suggests plausible candidate genes and offers some valuable insights into the molecular mechanisms underlying inter-individual variability in Li response. For example, renalase (*RNLS*) was one of the most highlighted genes in our study. In addition to its link to Li response in BP (Amare et al. 2023), serum renalase levels have been reported to be lower in patients with schizophrenia (SCZ) than in control individuals (Catak et al. 2019), and Li response was previously shown to inversely associate with the genetic risk for SCZ (Amare et al. 2018). *RNLS* is thought to modulate blood pressure and cardiac function, and has been associated with metabolic and cardiovascular alterations as well as kidney disease (Vijayakumar & Mahapatra 2022), all of which are affected by Li. Similar are the cases of *CSMD2* and *GRIN2A*, which are involved in the control of the complement cascade and N-methyl-D-aspartate (NMDA) receptor activity, respectively. Polymorphisms in both genes have also been associated with SCZ (Tang et al. 2006; Håvik et al. 2011) and their respective functions are reported targets of Li effects (Ghasemi & Dehpour 2011; Yu et al. 2015).

The investigation of how Li response measured by the Alda scale and Li response genes associate with the genetic predisposition to comorbid (medical) conditions is an important strength of our study. To our knowledge, this has not been investigated before. A high rate of medical comorbidity in BP, including cardiometabolic conditions, thyroid and kidney disease, is associated with worse clinical presentation and course, as well as higher mortality and increased socioeconomic burden (Sylvia et al. 2015). Although the risk of comorbidity can be exacerbated by pharmacological treatment, as discussed above, Li has shown beneficial effects on various systems. Therefore, it becomes crucial to gain a better understanding of the relationship between the effects of Li and medical comorbidity in BP. In this context, even when our PGS analyses resulted in only nominally significant findings, these suggested that common genetic factors link Li response and other conditions, particularly insomnia, in BP, and pinpointed potential contributing genes. In BP, sleep disturbances, from which the most frequent is insomnia, are not only highly prevalent, but an important predictor of quality of life, mood swings, suicide attempts, cognitive function and relapse rates (Steardo et al. 2019). Therefore, our observations might have implications for the prediction of Li response in BP patients as well as for disease management. Nevertheless, more studies will be required.

## Conclusions

Taken together, our findings suggest that the effects of Li on symptomatology and comorbidity in BP are partially modulated by common genetic factors, with differential effects between BP-I and BP-II. These findings might pave the way towards the development of more personalized treatment strategies for patients with BP.

### Supplementary Information

Supplementary Material 1.

## Data Availability

The data that support the findings of this study are available from ConLiGen, but restrictions apply to their availability.

## Abbreviations

AAO: Age at disease onset
BP: Bipolar disorders
ConLiGen: Consortium on Lithium Genetics
Li: Lithium
PGS: Polygenic score
SNP: Single nucleotide polymorphism
